# A Double Decomposition of Standard Deviation Below the Modal Age at Death and the Role of Causes of Death

**DOI:** 10.1007/s10680-025-09762-6

**Published:** 2026-04-10

**Authors:** Viorela Diaconu, Virginia Zarulli, Stefano Mazzuco

**Affiliations:** 1 Department of Statistical Sciences, University of Padova, Padova, Italy; 2 Present Address: Vienna Institute of Demography, Austrian Academy of Sciences, Postsparkasse, Dominikanerbastei 16, 1010 Vienna, Austria

**Keywords:** Lifespan variation, Modal age at death, Decomposition method, Causes of death, Europe

## Abstract

Lifespan inequality is a fundamental indicator of population health, reflecting inequalities in the timing of death. Life expectancy-based indicators have been widely used to monitor changes in lifespan variation across populations. This study proposes using indicators relative to the modal age at death (*M*): the standard deviation below the mode, $SD(M-)$, which captures variation in premature mortality, and the standard deviation above the mode, $SD(M+)$, which reflects variation in senescent mortality. Although trends in $SD(M+)$ are relatively well documented, less is known about how $SD(M-)$ has changed over time and what drives these changes. This study aims to (1) document and compare trends in $SD(M-)$ and $SD(M+)$ across high-income countries since 1960, and (2) examine the contribution of cause-specific mortality to changes in $SD(M-)$ in selected countries. To achieve this, we propose a novel two-step decomposition method. In the first step, changes in $SD(M-)$ are decomposed into two components: one attributable to shifts in the modal age itself (“mode” component) and another to changes in the shape of the age-at-death distribution (“distribution” component). In the second step, the “distribution” component is further decomposed by cause of death. Applying this framework to data from Japan and the U.S., results revealed that the decline in $SD(M-)$ in the U.S. was primarily driven by reductions in heart disease and neoplasm mortality. However, these gains were partially offset by increased variation linked to infectious diseases and external causes. In Japan, declines in $SD(M-)$ were primarily driven by reductions in cerebrovascular diseases, heart disease (women), and neoplasms (men), while increases in variation since the mid-1990s were largely attributable to external causes and neoplasms (women). This decomposition is a useful tool for identifying the factors that drive or hinder the compression of premature mortality.

## Introduction

Over the past 150 years, survival rates have improved dramatically in most countries around the world (World Health Organization, 2000), with life expectancy more than doubling in many populations (Oeppen & Vaupel, 2002). This remarkable progress has been accompanied by a significant reduction in lifespan variation, defined as inequality in the timing of death among individuals (Vaupel et al., 2011). When estimated from period life tables, lifespan variation reflects disparities in ages at death among individuals in a synthetic cohort exposed to current mortality rates. At the individual level, reduced lifespan variation implies a more predictable length of life. At the population level, it signals decreasing heterogeneity in health and mortality outcomes (Ehrlich, 2000; Edwards & Tuljapurkar, 2005; Edwards, 2013).

A growing body of literature has examined levels and trends in length of life variation to better understand how lifespan inequalities have evolved over time and across populations (e.g., Aburto et al. Aburto et al. (2018, 2020); Edwards and Tuljapurkar Edwards and Tuljapurkar (2005); Permanyer and Scholl Permanyer and Scholl (2019); Smits and Monden Smits and Monden (2009); Vaupel et al. Vaupel et al. (2011); Vigezzi et al. Vigezzi et al. (2022)). These studies revealed that low- and middle-income countries tend to exhibit higher lifespan inequality compared to high-income countries (Edwards & Tuljapurkar, 2005; Smits & Monden, 2009). Within countries, large differences are also evident across geographic areas: regions marked by higher levels of deprivation typically show greater lifespan variation than more affluent areas (Seaman et al., 2019; Su et al., 2024). Within the same country or region, inequalities stem from differences between genders and social groups. Men generally experience greater variation in age at death than women, and individuals from lower socioeconomic backgrounds, defined by education, income, and occupation, face more uncertainty about their lifespan compared to those from more advantaged groups (Diaconu et al., 2022; Sasson, 2016; van Raalte et al., 2014, 2018, 2011).

While the literature examining inequalities in all-cause mortality is extensive, there are relatively few studies that specifically analyze how causes of death contribute to fluctuations in these inequalities. Research that has explored this area indicates that circulatory diseases, perinatal and congenital diseases, and external causes contributed to the decline in lifespan inequality since 1980, with perinatal and congenital diseases playing the most significant role. In contrast, neoplasms were found to contribute to the increase in overall lifespan inequality (Permanyer & Vigezzi, 2024). At the subgroup level, cause-specific analyses have revealed that sex differences in lifespan variation are largely due to higher external mortality rates among men aged 30–39 years (Zazueta-Borboa et al., 2023), while alcohol- and smoking-related diseases have been key factors in widening lifespan variation between income groups in Finland (Tarkiainen et al., 2024).

Most of these studies have relied on dispersion measures relative to life expectancy, such as the standard deviation or Gini coefficient. Comparatively fewer studies have utilized dispersion indicators centered on the modal age at death (*M*), the most frequent age at death, since its introduction to contemporary demography by Kannisto (Kannisto, 2001). Among mode-based measures, the standard deviation above the mode, $SD(M+)$, has been the most widely applied, primarily because of its utility in monitoring changes in mortality dispersion at older ages. These studies have documented increasing mortality compression at older ages, suggesting a reduction in lifespan inequality among the elderly in several high-income countries (Cheung et al., 2005, 2008, 2009; Cheung & Robine, 2007; Kannisto, 2001, 2007; Ouellette & Bourbeau, 2011). In contrast, measures of dispersion below the mode, $SD(M-)$, and around the mode, *SD*(*M*), have received considerably less attention, despite their potential to capture important inequality dynamics at younger and middle ages.

This study therefore focuses on trends in lifespan inequality centered on the modal age at death, adding to the limited research that examines these trends through the lens of cause-specific mortality. The reason being that, the modal age at death presents several advantages over traditional life expectancy at birth or at other ages (e.g., 50 or 65). First, it is not influenced by changes in infant and child mortality (Horiuchi et al., 2013). Second, it allows for the distinction between premature and senescent mortality without relying on arbitrary age cutoffs (Kannisto, 2001). Additionally, it provides a more accurate reflection of survival improvement, particularly when these improvements are primarily driven by reductions in mortality at older ages (Canudas-Romo, 2010; Horiuchi et al., 2013; Kannisto, 2001). Finally, it allows comparing individuals with similar survival chances across time and countries (Diaconu et al., 2022). Of the available mode-based dispersion indicators, we selected $SD(M-)$. Our focus on $SD(M-)$ is supported by a comparative analysis of long-term trends in $SD(M-)$ and $SD(M+)$ across various European and non-European countries. To our knowledge, this type of empirical examination has not been conducted previously. This comparison revealed that $SD(M-)$ exhibits greater heterogeneity across countries and genders than $SD(M+)$, making it a more informative indicator for examining differences in lifespan variation across populations .

To analyze changes in $SD(M-)$ over time, we introduce a novel two-step decomposition method. In the first step, we partition changes in $SD(M-)$ between two calendar years into two distinct components: a “distribution” component, capturing transformations in the distribution of deaths below the modal age, and a “mode” component, reflecting changes in the modal age itself. Changes in the distribution of deaths below the mode may result from shifts in the timing of deaths (e.g., earlier or later deaths), transformations in the distribution’s shape (e.g., increased dispersion or skewness), variations in the overall mortality level, or a combination of these factors. This first step allows isolating the effects of the changing modal age, as increases in M can automatically lead to higher values of $SD(M-)$. This decomposition provides valuable insights into changes in mortality inequality over time, highlighting the relative contributions of distributional transformations and shifts in the modal age at death. By doing so, it allows for the identification of which of these components has been the primary driver of changes in $SD(M-)$ over time. In the second step, we further decompose the “distribution” component by specific causes of death. This allows us to identify the causes that contributed most to these changes and provides insight into the factors driving inequalities in premature mortality.

To demonstrate the utility of this dual decomposition approach, we apply it to data from Japan and the United States. We have decided to focus solely on the U.S. and Japan for two reasons: (1) our earlier analysis showed that these two countries exhibit the most divergent long-term trends in $SD(M-)$, making them ideal for illustrating the potential of the decomposition, and (2) cause-of-death data for these countries have been harmonized to account for changes in ICD coding over time, reducing the risk of bias. Although the method can be applied to other countries, including European ones, our goal here is to demonstrate the utility of the framework using two well-documented and contrasting cases. We hope this inspires future applications in other settings.

This article is structured as follows. Section 2 introduces the dispersion indicators $SD(M-)$, $SD(M+)$, and *SD*(*M*). Sections 3.1 and 3.2 focus on the double decomposition of changes in $SD(M-)$. Section 4 describes the methods used to estimate the mortality function for calculating the dispersion indicators. The results are presented in Sect. 5, followed by a discussion in Sect. 6.

## Dispersion Indicators Relative to *M*

For a given calendar year, *t*, the standard deviation below the mode, $SD(M-)$, informs on the concentration of deaths at ages *below* the modal age at death *M*, and is obtained as follows:

1 $$\begin{aligned} SD(M-)_t = \sqrt{ \frac{\int \limits _{x_0}^{M_t} (x-M_t)^2 \> f_t(x) \> dx}{\int \limits _{x_0}^{M_t} f_t(x) \> dx} } \end{aligned}$$

where *x* is the age at death, $x_0$ is the initial value of *x* (usually $x_0>0$ to disregard infant mortality), and $f_t(.)$ the distribution of ages at death at time *t*.

The other two dispersion indicators relative to *M*, are the standard deviation above the mode, $SD(M+)$ which reflects the concentration of deaths at ages above *M*, and the standard deviation around the mode, *SD*(*M*), which measures the dispersion of deaths centered at the modal age over the entire age range. They are defined as:

2 $$\begin{aligned} SD(M+)_t = \sqrt{ \frac{\int \limits _{M_t}^\omega (x-M_t)^2 \> f_t(x) \> dx}{\int \limits _{M_t}^\omega f_t(x) \> dx} } \qquad SD(M)_t = \sqrt{ \frac{\int \limits _{x_0}^\omega (x-M_t)^2 \> f_t(x) \> dx}{\int \limits _{x_0}^\omega f_t(x) \> dx} }. \end{aligned}$$

Variation in deaths at ages below the mode reflects inequality in premature mortality, which is defined as deaths occurring between ages 10 and those corresponding to senescent mortality. According to Lexis (1877), these ages are centered around the modal age at death. In turn, variatation in ages at death around and above the mode captures inequality in senescent mortality.

The modal age at death in calendar year *t*, denoted as $M_t$, is defined as the age at which the highest proportion of deaths occurs. It is obtained by maximizing the density function, $f_t(x)$, for year *t*. That is,

3 $$\begin{aligned} M_t = \max _x \> f_t(x). \end{aligned}$$

The density function, $f_t(x)$, is obtained by multiplying the force of mortality $\mu _t(x)$ by the survival function $S_t(x)$, as:

4 $$\begin{aligned} f_t(x) = \mu _t(x)S_t(x) = \mu _t(x) \exp \Big [- \int _{0}^{x} \mu _t(u)du\Big ]. \end{aligned}$$

From Eq. (4) the density function can be expressed in terms of the force of mortality alone. This is convenient as in this way we can derive the distribution of deaths by age from the smoothed estimate of $\mu (x)$.

## Decomposition of Changes in $SD(M-)$

### First Level: “Mode” and “Distribution” Components

According to Eq. (1), changes in the standard deviation indicators are driven by two components: the central value, *M*, and the density function, *f*(*x*). The influence of each component can be examined independently by holding the other constant. When *M* increases while *f*(*x*) remains unchanged, $SD(M-)$ increases and $SD(M+)$ decreases, as *M* shifts away from the left tail and closer to the right tail of the age-at-death distribution. The net effect on *SD*(*M*) depends on the relative strength of these opposing changes. In contrast, the impact of changes in the age-at-death distribution when *M* is held constant is less straightforward. This is because the proportion of deaths may increase or decrease at different ages.

**Fig. 1:**
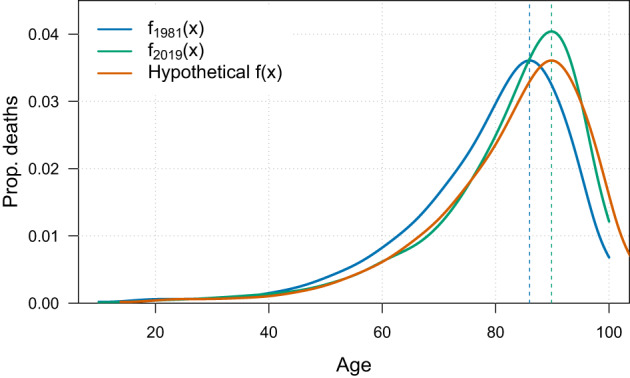
P-spline-smoothed age-at-death distributions, ${\hat{f}}(x)$, for U.S. women in 1981 (blue) and 2019 (green), along with a hypothetical distribution (orange). Note: Smoothing was based on ages above 10. Source: (HMD, 2024)

Figure 1 illustrates this decomposition by showing the P-spline-smoothed age-at-death distributions for U.S. women for two calendar years: 1981 (blue curve) and 2019 (green curve). The orange curve represents a hypothetical distribution in which only the modal age at death (*M*) shifts from 1981 to 2019, while the overall shape of the distribution remains fixed at its 1981 form. Changes in $SD(M-)$ between 1981 and 2019 can therefore be decomposed into two components: one due to changes in the modal age at death (the “mode” component), and one due to changes in the overall shape of the age-at-death distribution (the “distribution” component). The effect of the “mode” component is captured by the difference in $SD(M-)$ between the blue and orange curves. The effect of the “distribution” component is represented by the difference in $SD(M-)$ between the orange and green curves. The $SD(M-)$ of the orange curve is calculated using the age-at-death distribution of 1981 combined with the 2019 modal age at death.

In general, changes in $SD(M-)$ between two calendar years $t_1$ and $t_2$ can be expressed as:

5 $$\begin{aligned} \Delta SD(M-)= & SD(M-)_{t_2} - SD(M-)_{t_1} =\nonumber \\= & \sqrt{ \frac{\int \limits _{x_0}^{M_{t_2}} (x-M_{t_2})^2 \> f_{t_2}(x) \> dx}{\int \limits _{x_0}^{M_{t_2}} f_{t_2}(x) \> dx} } - \sqrt{ \frac{\int \limits _{x_0}^{M_{t_1}} (x-M_{t_1})^2 \> f_{t_1}(x) \> dx}{\int \limits _{x_0}^{M_{t_1}} f_{t_1}(x) \> dx}} \end{aligned}$$

By adding and subtracting $\sqrt{ \frac{\int \limits _{x_0}^{M_{t_2}} (x-M_{t_2})^2 \> f_{t_1}(x) \> dx}{\int \limits _{x_0}^{M_{t_2}} f_{t_1}(x) \> dx} }$ to Eq. (5), the change in $SD(M-)$ can be rewritten as:

6 $$\begin{aligned} \begin{aligned} \Delta SD(M-) = {}&\underbrace{ \sqrt{ \frac{\int \limits _{x_0}^{M_{t_2}} (x-M_{t_2})^2 \> f_{t_2}(x) \> dx}{\int \limits _{x_0}^{M_{t_2}} f_{t_2}(x) \> dx} } - \sqrt{ \frac{\int \limits _{x_0}^{M_{t_2}} (x-M_{t_2})^2 \> f_{t_1}(x) \> dx}{\int \limits _{x_0}^{M_{t_2}} f_{t_1}(x) \> dx} }}_{\Delta f(x)} + \\ +&\underbrace{\sqrt{ \frac{\int \limits _{x_0}^{M_{t_2}} (x-M_{t_2})^2 \> f_{t_1}(x) \> dx}{\int \limits _{x_0}^{M_{t_2}} f_{t_1}(x) \> dx} } - \sqrt{ \frac{\int \limits _{x_0}^{M_{t_1}} (x-M_{t_1})^2 \> f_{t_1}(x) \> dx}{\int \limits _{x_0}^{M_{t_1}} f_{t_1}(x) \> dx}} }_{\Delta M} \end{aligned} \end{aligned}$$

where $\Delta f(x)$ is the change in $SD(M-)$ due solely to the change in *f*(*x*) and $\Delta M$ the change in $SD(M-)$ due solely to the change in *M*. This decomposition method can also be applied to $SD(M+)$ and *SD*(*M*). However, the upper and lower bounds of the integral must be adjusted accordingly.

We are interested in identifying the causes of death responsible for the difference between the two curves at ages below *M*. To do this, we further decompose the “distribution” component by cause of death, which is presented in the next section.

### Second Level: “Distribution” Component by Cause of Death

Changes in the all-cause distribution of ages at death result from shifts in the distribution of cause-specific ages at death. To identify which causes of death were responsible for the changes in $SD(M-)$ over time, we decompose the first two terms of Eq. (6) by cause of death.

The all-cause density function is the product of the all-cause force of mortality and the survival function (Eq. 4). Assuming that the causes of death are mutually exclusive and mutually exhaustive (Preston, Heuveline, and Guillot 2001), the all-cause force of mortality can be obtained by summing the cause-specific forces of mortality. The all-cause density function can thus be rewritten as:

7 $$\begin{aligned} f(x)&= \mu (x)S(x) \end{aligned}$$

8 $$\begin{aligned}&= (\mu ^1(x) + \mu ^2(x) + \ldots + \mu ^K(x))S(x) \end{aligned}$$

9 $$\begin{aligned}&= f^1(x) + f^2(x) + \ldots + f^K(x) \end{aligned}$$

where *K* represents the total number causes of death.

Replacing *f*(*x*) with its expression in Eq. (10) and rearranging the terms yields the following:

10 $$\begin{aligned} \begin{aligned} \Delta SD(M-) = {}&\sqrt{ \frac{\int \limits _{x_0}^{M_{t_2}} (x-M_{t_2})^2 \>\Bigl ( f^1_{t_2}(x) + f^2_{t_2}(x) + \ldots + f^k_{t_2}(x) \Bigr ) \> dx}{\int \limits _{x_0}^{M_{t_2}} f_{t_2}(x) \> dx} } - \\&\sqrt{ \frac{\int \limits _{x_0}^{M_{t_2}} (x-M_{t_2})^2 \> \Bigl ( f^1_{t_1}(x) + f^2_{t_1}(x) + \ldots + f^k_{t_1}(x) \Bigr ) \> dx}{\int \limits _{x_0}^{M_{t_2}} f_{t_1}(x) \> dx} } \\ \end{aligned} \end{aligned}$$

Since the square root of a difference is not equal to the difference of the square roots, we will examine the changes in variance below the mode, $V(M-)$, rather than $SD(M-)$. This mathematical transformation only changes the scale. As Figs. 12 and 13 in the Appendix show, the underlying patterns and trends of $SD(M-)$ resemble those of $V(M-)$ over time and across countries.

Hence,^1^

11 $$\begin{aligned} \begin{aligned} \Delta V(M-) = {}&\underbrace{\frac{\int \limits _{x_0}^{M_{t_2}} (x-M_{t_2})^2 \> f^1_{t_2}(x) \> dx}{\int \limits _{x_0}^{M_{t_2}} f_{t_2}(x) \> dx} - \frac{\int \limits _{x_0}^{M_{t_2}} (x-M_{t_2})^2 \> f^1_{t_1}(x) \> dx}{\int \limits _{x_0}^{M_{t_2}} f_{t_1}(x) \> dx} }_{\Delta f^1(x)} + \\ + {}&\underbrace{ \frac{\int \limits _{x_0}^{M_{t_2}} (x-M_{t_2})^2 \> f^2_{t_2}(x) \> dx}{\int \limits _{x_0}^{M_{t_2}} f_{t_2}(x) \> dx} - \frac{\int \limits _{x_0}^{M_{t_2}} (x-M_{t_2})^2 \> f^2_{t_1}(x) \> dx}{\int \limits _{x_0}^{M_{t_2}} f_{t_1}(x) \> dx}} _{\Delta f^2(x)} + \ldots + \\ + {}&\underbrace{\frac{\int \limits _{x_0}^{M_{t_2}} (x-M_{t_2})^2 \> f^k_{t_2}(x) \> dx}{\int \limits _{x_0}^{M_{t_2}} f_{t_2}(x) \> dx} - \frac{\int \limits _{x_0}^{M_{t_2}} (x-M_{t_2})^2 \> f^k_{t_1}(x) \> dx}{\int \limits _{x_0}^{M_{t_2}} f_{t_1}(x) }} _{\Delta f^k(x)} \end{aligned} \end{aligned}$$

The cause-specific components can be interpreted as follows: if $\Delta f^k(x) < 0$ then changes in $f^k(x)$ at ages below *M* lead to a reduction in $V(M-)$ between $t_1$ and $t_2$, while if $\Delta f^k(x) > 0$, changes in $f^k(x)$ at ages below *M* lead to an increase in $V(M-)$ between $t_1$ and $t_2$.

## Estimation of the Mortality Functions and the Dispersion Indicators

As seen in Eqs. (3), (4), and (7), the modal age at death, *M*, and the all-cause density functions, *f*(*x*), depend solely on the estimation of the all-cause and force of mortality, $\mu _t(x)$. In turn, the cause-specific density functions are estimated from the cause-specific force of mortality.

### All-Cause Force of Mortality

We use observed deaths from all causes of death and population exposures by single-year of age, sex, and calendar year from the Human Mortality Database (2024). We analyze data from 21 countries, spanning from 1960 to 2019, grouped into four regions: Northern Europe (Denmark, Finland, Norway, Sweden), Western Europe (Austria, Belgium, France, Ireland, Netherlands, UK), Eastern Europe (Estonia, Hungary, Latvia, Lithuania, Poland), and Outside Europe (Australia, Canada, Japan, New Zealand, U.S.).

For a given calendar year, the force of mortality, $\mu _t(x)$, is generally estimated by the central death rate obtained from observed deaths and exposures by single-year of age. Although this straightforward technique is quite simple, it does not allow to maintain the continuity of the age patterns of mortality presented in Eq. (4). To overcome these limitations, we smooth the age-specific death rates with penalized *B*-splines, (*P*-splines) for each country, calendar year, and sex. This allows to model the force of mortality as a combination of *B*-splines basis and penalized coefficients. With a smooth estimate of $\mu (x)$ we can use formula (4) to derive a smooth estimate of *f*(*x*), from which we can estimate the value of *M*.

The *B*-spline smoothing technique ensures flexibility and accuracy, while the penalty, acting on the coefficients of adjacent *B*-splines, ensures the smoothness of the fit (Camarda, 2012; Currie et al., 2004). The balance between smoothness and precision is controlled by a smoothing parameter, selected using the Bayesian Information Criterion (BIC) (Schwarz, 1978), which has been shown to be the most suitable for mortality data (Currie et al., 2004). The smoothing procedure is applied to mortality data from age 10 and above. We exclude child and infant mortality from our analysis because they have unique characteristics that require the use of smoothing methods tailored to this type of data (Ouellette & Bourbeau, 2011; Camarda, 2008; Krivobokova et al., 2010).

### Cause-Specific Force of Mortality

Data on causes of death are taken from the Human Cause-of-Death Database, which is part of the Human Mortality Database, ensuring consistency and reliability between the all-cause and cause-specific data sources. In this dataset, observed deaths by cause are available for 5-year age groups, sex, and calendar year, while population exposure counts are provided by single year of age, sex, and calendar year. The database includes both the reconstructed and original classification series of causes of death. The reconstructed series, which classifies causes according to a fixed classification that takes into account changes in the International Classification of Diseases (ICD), eliminates the biasing effects of successive ICD revisions. This series is available for a subset of countries in the HMD, with different years of coverage for each country. In this dataset, causes of death are classified according to long ($\sim$ 200 categories), intermediate ($\sim$ 50 categories), and short ($\sim$ 15 categories) lists of causes of death, which are the same for all countries.

To obtain the cause-specific forces of mortality and to ensure the equality in Eq. (7) - that is, the all-cause force of mortality is equal to the sum of the cause-specific forces of mortality - we apply the Penalized Composite Link Model (PCLM) approach. The PCLM has previously been used to estimate continuous data from grouped observations (Camarda, 2012; Rizzi et al., 2015; Lambert & Eilers, 2009; Permanyer et al., 2018; Aburto et al., 2018; Riffe & Acosta, 2021; Torres et al., 2019). This method allows for the disaggregation of 5-year death rates into 1-year death rates, which can then be used to estimate the latent force of mortality using penalized B-splines. The smooth cause-specific density functions are then derived by multiplying the cause-specific mortality forces by the B-spline coefficients.

Once the death rates have been smoothed, the continuous all-cause and cause-specific forces of mortality, ${\hat{\mu }}_t(x)$ and ${\hat{\mu }}^k_t(x)$, can be estimated at any desired level of precision using penalized *B*-splines. To obtain the smooth force of mortality for each cause of death, we use the estimated *B*-spline coefficients and evaluate the corresponding B-splines on a fine grid. The all-cause smooth density function, ${\hat{f}}(x)$, is obtained from Eq. (4), with ${\hat{S}}(x)$ being derived from ${\hat{\mu }}(x)$ using the Riemann sum integration technique. The all-cause modal age at death, ${\hat{M}}$, can then be obtained with high numerical precision from the all-cause smooth density function. Finally, the standard deviation indicators relative to *M* are computed using Eqs. (1) and (2).

For the decomposition by cause of death, we focus on Japan and the U.S. Death counts by cause of death are available for these countries from 1979–2021 (Japan) and 1981–2021 (U.S.), but our analysis is restricted for the common data period for both: 1981-2019. Of the three available cause-of-death classifications, we selected the short list, as a more detailed classification could result in small numbers of deaths for specific causes at certain ages. This could affect the accuracy of data disaggregation by single year of age. The selected causes of death categories are presented in Table 1.

**Table 1:** Selected causes of death and their respective ICD10 codes

ICD10 code	Cause of death
A00-B99	Certain infectious diseases
C00-D48	Neoplasms
E00-E88	Endocrine, nutritional, and metabolic diseases
F01-F99	Mental and behavioural diseases
I00-I51	Heart diseases
G45, I60-I69	Cerebrovascular disease
J00-J22, U04, U07-U10	Acute respiratory diseases
K00-K63, K65-K92	Diseases of the digestive system
V01-Y89	External causes
	*Rest of causes*
*D50-D89*	*Diseases of the blood and blood-forming organs*
*G00-G44, G47-H93*	*Diseases of the nervous system and the sense organs*
*I70-I99, K64*	*Other and unspecified disorders of the circulatory system*
*J30-J98*	*Other respiratory diseases*
*L00-M99*	*Diseases of the skin and subcutaneous tissue, musculoskeletal system and connective tissue*
*N00-O99*	*Diseases of the genitourinary system and complications of pregnancy, childbirth and puerperium*
*P00-Q99, R95*	*Certain conditions originating in the perinatal period and congenital malformations/anomalies*

## Results

### Trends in $SD({\hat{M}}-)$ and $SD({\hat{M}}+)$

**Fig. 2:**
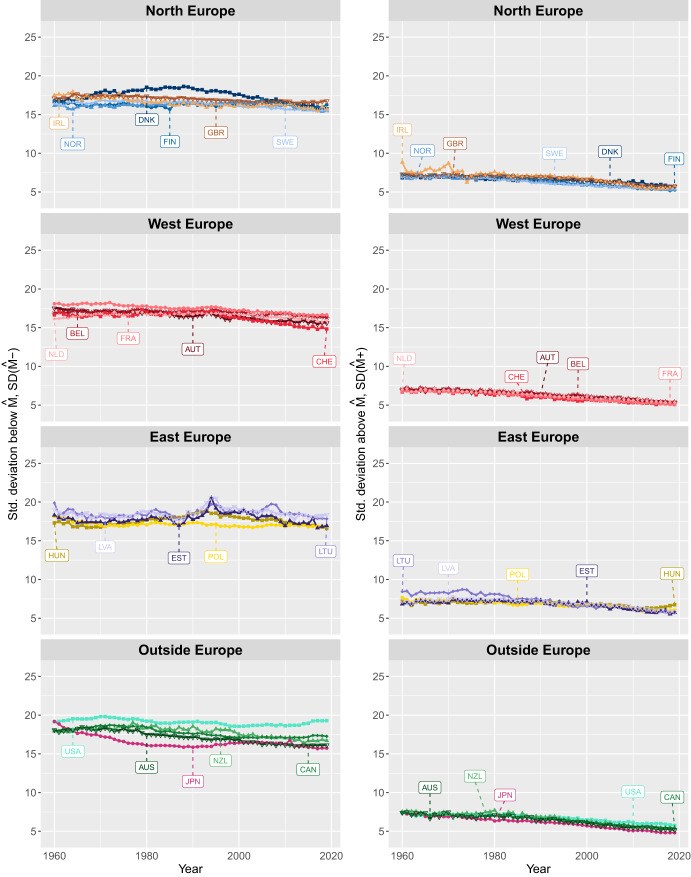
Trends in standard deviation below, $SD({\hat{M}}-)$, and above, $SD({\hat{M}}+)$, the modal age at death for women in selected European and non-European countries, 1960 to 2019. Note: Smoothing was based on ages above 10. Source: HMD (2024)

**Fig. 3:**
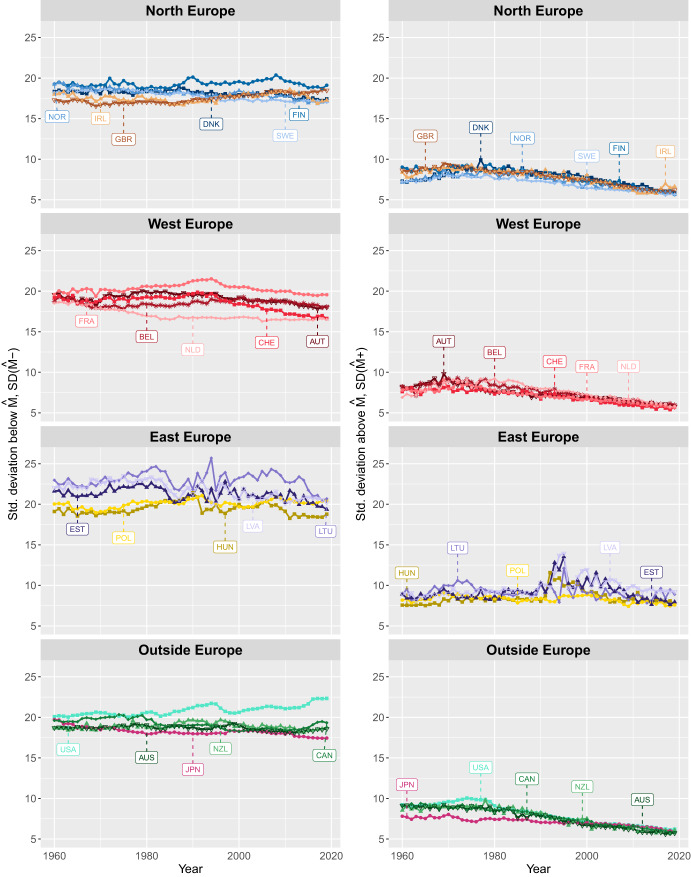
Trends in standard deviation below, $SD({\hat{M}}-)$, and above, $SD({\hat{M}}+)$, the modal age at death for men in selected European and non-European countries, 1960 to 2019. Note: Smoothing was based on ages above 10. Source: HMD (2024)

Figures 2, 3 show trends in the variability of deaths at ages *below* and *above* *M* for women and men in several European and non-European countries since 1960. As shown, $SD({\hat{M}}-)$ levels are consistently higher than $SD({\hat{M}}+)$ across sexes, calendar years, and countries, indicating greater variability in deaths at ages *below* *M* than *above* it. The patterns of trends in $SD({\hat{M}}-)$ and $SD({\hat{M}}+)$ exhibit very different trajectories over time. In general, variability of ages at death below ${\hat{M}}$ has declined for both men and women in all the countries studied since 1960. However, this decline was interrupted by periods of increasing variability, which occurred at different time periods across countries. In contrast, variability of age at death above *M* has steadily and consistently declined since the late 1900 s. Furthermore, there are greater differences across countries in the variability of deaths at ages below than above *M*, for both men and women.

A closer examination of the results for women reveals that in 1960, the standard deviation of ages at death below the modal age ($SD({\hat{M}}-)$) varied significantly by country. It ranged from 19 years in Japan, Lithuania, Latvia, and the U.S. to approximately 16 years in Norway, Switzerland, and the Netherlands. By 2019, women in the U.S. recorded the highest $SD({\hat{M}}-)$, reaching about 19 years, while Swiss women had the lowest at around 15 years. Among Northern European countries, Denmark consistently showed larger $SD({\hat{M}}-)$ values (approximately 17 years) compared to the remaining countries in this group, particularly from the 1970 s to the mid-2000 s. France and the U.S. showed the highest variability in deaths occurring at ages below the mode compared to the other countries in their regions. In Eastern Europe, both Latvia and Lithuania had the highest $SD({\hat{M}}-)$ values.

At ages above the mode, Ireland and Lithuania recorded the highest values for women in 1960, with figures close to 9 years and 8.5 years respectively. In other countries, $SD({\hat{M}}+)$ generally fluctuated around 7 years. By 2019, Hungary reported the largest $SD({\hat{M}}+)$ at nearly 7 years, while the remaining countries exhibited values ranging from 6.0 years in Poland to 4.8 years in Japan.

The comparison of $SD({\hat{M}}-)$ levels across countries within the same region shows larger differences in 2019 than in 1960 outside Europe, similar differences in Western and Northern Europe, and a slight decline in Eastern Europe. In contrast, $SD({\hat{M}}+)$ trends reveal smaller differences across countries by the end of the study period compared to the beginning.

Among men, the highest $SD({\hat{M}}-)$ in 1960 was observed in Lithuania, at around 21 years, while British men had the lowest, about 18 years. In most countries, $SD({\hat{M}}-)$ increased during the period 1970-1990 followed by a decline in the following years. By 2019, $SD({\hat{M}}-)$ levels were lower than in 1960 for most countries, with the exception of Poland, the UK, and the U.S. The latter two countries experienced the highest increase of about one and two years, respectively. In contrast, Estonia, Japan, Lithuania, the Netherlands, Norway, and Switzerland saw a reduction of about 2 years in the variation of age at death between 1960 and 2019. At the end of the study period, the highest $SD({\hat{M}}-)$ is observed for U.S. men (around 22 years) and the lowest in Switzerland and the Netherlands (around 16 years). Differences in $SD({\hat{M}}-)$ between countries were greater in 2019 than in 1960, particularly in Western Europe and Outside Europe.

In contrast to $SD({\hat{M}}-)$, the differences between countries in $SD({\hat{M}}+)$ were greater at the beginning of the study period than at the end. Over time, $SD({\hat{M}}+)$ has converged to similar levels across all countries, with the exception of Eastern Europe. In 1960, the highest $SD({\hat{M}}+)$, around 9 years, was observed in several countries, including Australia, Estonia, Canada, Finland, Latvia, Lithuania, and the U.S.. Meanwhile, Denmark, the Netherlands and Sweden recorded the lowest values, around 7 years. In 2019, $SD({\hat{M}}+)$ ranged from 9 years in Latvia and Lithuania to just over 5.5 years in most other countries, with the exception of Estonia, Hungary, Ireland, New Zealand, Poland, the UK, and the U.S., which recorded slightly higher values.

In summary, since the 1960s, the dispersion of ages at death has decreased both below and above *M*. However, differences in age-at-death variability between countries are more pronounced below the mode than above it. In particular, while there were seizable differences in $SD({\hat{M}}+)$ at the beginning of the period, by the end of the 2010s, variability in age at death had converged to similar levels across countries. In contrast, country differences in $SD({\hat{M}}-)$ remained relatively stable over time, and even increased in some regions. Compared to $SD({\hat{M}}+)$, $SD({\hat{M}}-)$ shows greater variability across countries.

Trends in $SD({\hat{M}})$, which reflects the variability of ages at death both below and above the mode, closely mirror those in $SD({\hat{M}}-)$ for both men and women and over time across all the countries studied (see Fig. 11 in the Appendix). Additionally, the patterns of country differences for $SD({\hat{M}})$ are strikingly similar to those for $SD({\hat{M}}-)$, indicating that there are parallel trends in lifespan inequalities across countries. However, $SD({\hat{M}})$ consistently reaches higher values than $SD({\hat{M}}-)$, reflecting greater overall variability in deaths when considering the entire age range.

#### Relation Between $SD({\hat{M}}-)$ and $SD({\hat{M}}+)$

**Fig. 4:**
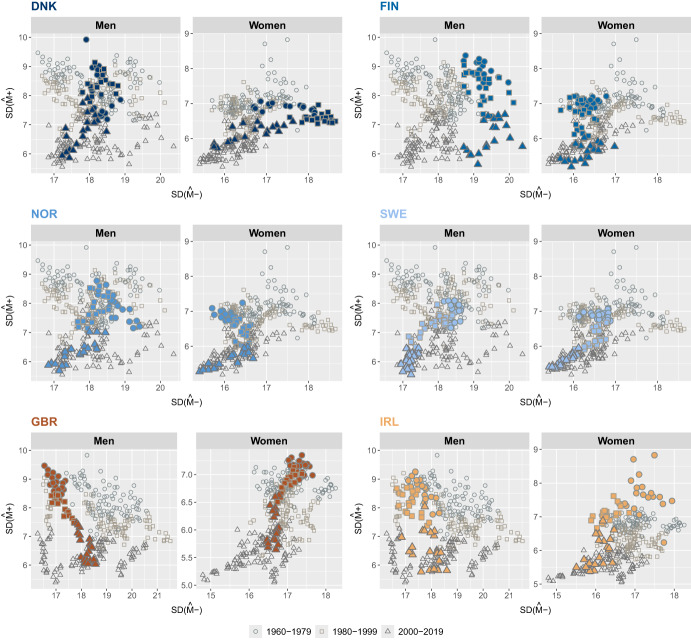
Joint evolution of the standard deviation below, $SD({\hat{M}}-)$, and above, $SD({\hat{M}}+)$, the modal age at death for men and women in Northern Europe, 1960-2019. Note: Smoothing was based on ages above 10. Source: HMD (2024)

**Fig. 5:**
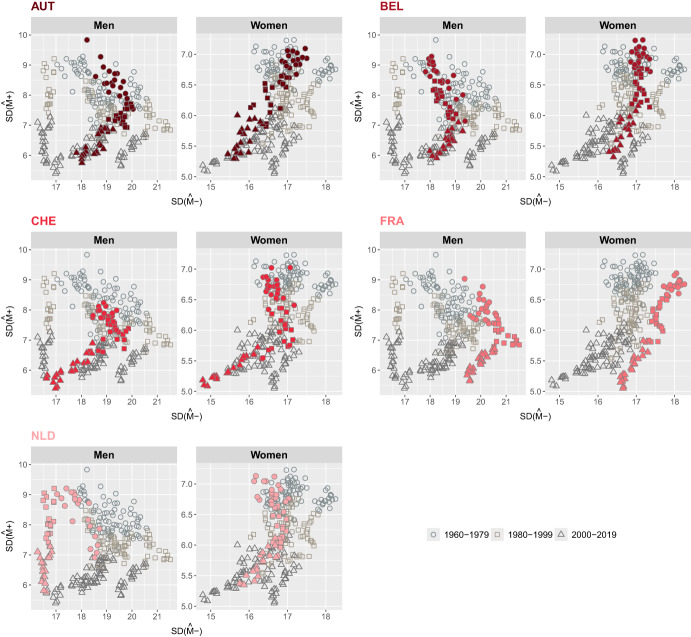
Joint evolution of the standard deviation below, $SD({\hat{M}}-)$, and above, $SD({\hat{M}}+)$, the modal age at death for men and women in Western Europe, 1960-2019. Note: Smoothing was based on ages above 10. Source: HMD (2024)

**Fig. 6:**
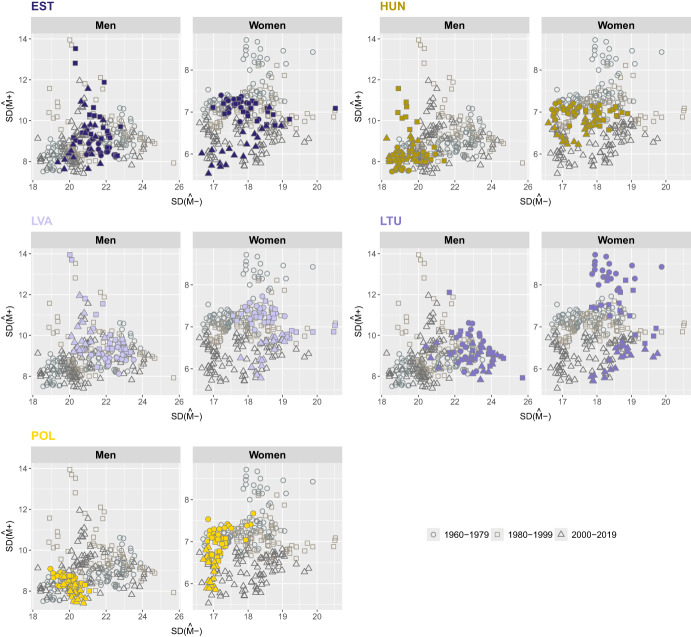
Joint evolution of the standard deviation below, $SD({\hat{M}}-)$, and above, $SD({\hat{M}}+)$, the modal age at death for men and women in Eastern Europe, 1960-2019. Note: Smoothing was based on ages above 10. Source: HMD (2024)

**Fig. 7:**
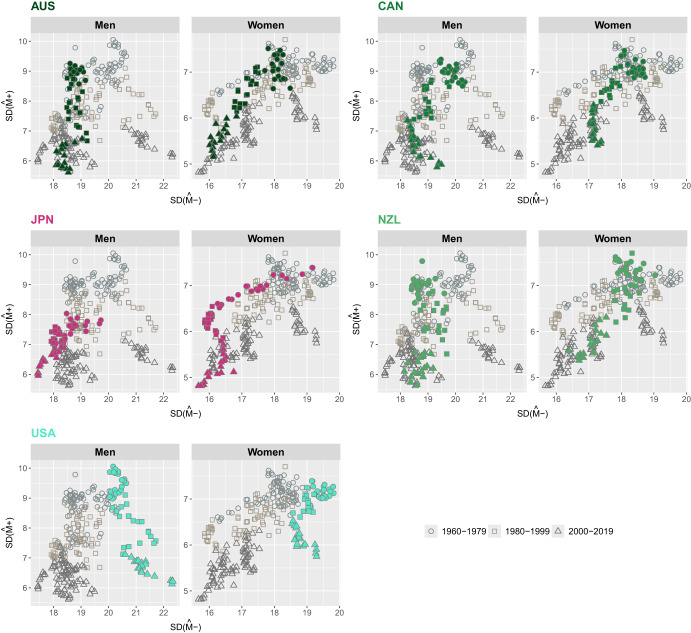
Joint evolution of the standard deviation below, $SD({\hat{M}}-)$, and above, $SD({\hat{M}}+)$, the modal age at death for men and women outside Europe, 1960-2019. Note: Smoothing was based on ages above 10. Source: HMD (2024)

Figures 4 to 7 illustrate the joint evolution of $SD({\hat{M}}-)$ and $SD({\hat{M}}+)$ for both men and women across the 21 countries included in the study from 1960 to 2019. Over the study period, $SD({\hat{M}}+)$ showed a decreasing trend in the countries studied. In contrast, $SD({\hat{M}}-)$ has followed different trajectories that vary over time, by gender, and across countries, and thus shows greater variability than $SD(M+)$.

In northern European countries, the dispersion of deaths at ages below the mode increased for Danish and Norwegian women up to the period 2000-2019 and remained relatively stable for Finnish and British women (Fig. 4). For men, $SD({\hat{M}}-)$ increased in the UK and to some extent in Ireland, remained relatively stable in Finland and Norway, and decreased slightly in Denmark and Sweden. Compared with the countries in this group, women in Denmark and men in Finland have the highest $SD({\hat{M}}-)$ values, while women in Ireland have the highest $SD({\hat{M}}+)$ values, especially in the period 1960-1979.

In western European countries, a downward trend in the variability of deaths at ages below the mode is observed for women throughout the study period and only after the 1980s (Fig. 5). Before the 1980s, $SD({\hat{M}}-)$ for men increased in all countries. France (men and women) has the highest $SD({\hat{M}})$ and the Netherlands (men) the lowest among the countries in this group.

In Eastern Europe, a decrease in $SD({\hat{M}}-)$ is observed for Latvian, Lithuanian and Polish women (Fig. 6). For Estonian women, the value of $SD({\hat{M}}-)$ remained fairly stable until the period 2000-2019 and then decreased slightly. Men exhibit similar trends in $SD({\hat{M}}-)$ in most of the countries in this group, showing a slight decrease over time. However, Polish men stand out, as their trend shows a slight increase since the 1960 s. Latvian and Lithuanian women had the highest values of $SD({\hat{M}}-)$, while Polish women had the lowest. Among men, the lowest variability of deaths at ages below the mode is observed for Hungarian men and the highest for Lithuanian men.

Outside Europe, most countries have experienced a decline in both $SD({\hat{M}}+)$ and $SD({\hat{M}}-)$ since the 1960 s, with the notable exception of the U.S., where variability below the mode has increased (Fig. 7). A closer look at the results shows that Japan and the U.S. have two opposite trends for $SD({\hat{M}}-)$ and a rather similar one for $SD({\hat{M}}+)$. Japanese women stand out for their sharp decline in $SD({\hat{M}}-)$ and for maintaining the lowest dispersion of deaths below the modal age. In contrast, U.S. men and women consistently have the highest $SD({\hat{M}}-)$ values.

The results presented in Figs. 4 to 7 show that, on average, both $SD({\hat{M}}-)$ and $SD({\hat{M}}+)$ decreased over time, pointing at a general process of compression of mortality. However, while $SD({\hat{M}}+)$ shows a downward trend for all countries (with the only exception of Eastern European countries), we can observe that, in several cases (e.g., women in Denmark, men and women in Finland, men in several Northern and Central European countries, both sexes in the U.S.), the evolution over time of $SD({\hat{M}}-)$ is more complex and, in several instances, even shows an increasing trend. This makes $SD({\hat{M}}-)$ a more informative indicator of lifespan variability compared to $SD({\hat{M}}+)$. We can particularly focus on the contrasting trends between Japan and the U.S., with $SD({\hat{M}}+)$ declining for both while $SD({\hat{M}}-)$ is declining for Japan and increasing for the U.S.This suggests that variability *below* the mode is more responsible for the noticeable differences in overall mortality between the two countries. For this reason, we will focus on this index of lifespan inequality for the rest of this work, even though the proposed decomposition technique can be applied equally well to $SD(M+)$.

### Decomposition of $SD(M-)$: the Case of Japan and the United States

#### First Level: “Mode” and “Distribution” Components

**Fig. 8:**
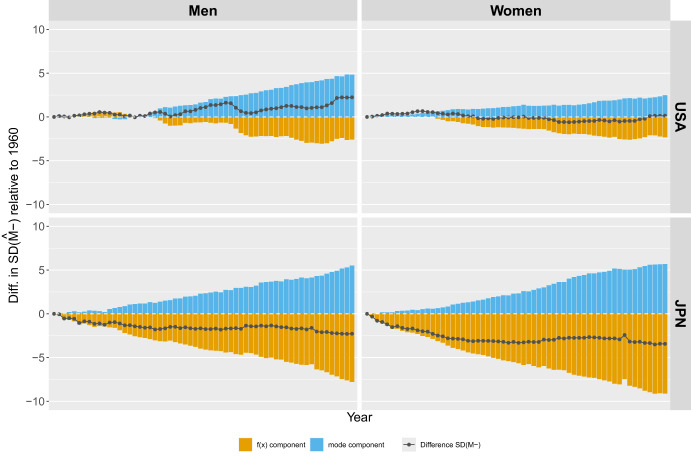
Annual difference in the standard deviation below the mode, $SD({\hat{M}}-)$, relative to 1961 and the contribution of the “mode” and “distribution” components for men and women in the U.S. and Japan. Note: Smoothing was based on ages above 10. Source: HMD (2024)

Figure 8 shows the annual differences in the standard deviation below the mode relative to the 1960 baseline (gray line) for both men and women in Japan and the U.S. The variability of deaths at ages below the mode decreased for both men and women in Japan, remained relatively stable for U.S. women, and increased for U.S. men since the 1980 s.

The decomposition of changes in $SD({\hat{M}}-)$ into “mode” and “distribution” components shows that it was primarily the shift of *M* to higher ages that drove the increase in $SD({\hat{M}}-)$ for U.S. men, rather than changes in the distribution of ages at death. In fact, changes in the “distribution” component led to a reduction in the variability of deaths at ages below *M*, reflecting improvements in survival from premature mortality. The slight increase in $SD({\hat{M}}-)$ for U.S. women in recent years is also due to the shift of *M* to higher ages rather than an increase in mortality at ages below *M*. In contrast to the U.S., the increase in $SD({\hat{M}}-)$ in Japan, driven by the increase in *M*, was completely offset by the decrease in the “distribution” component.

#### Second Level: Causes of Death Components

**Fig. 9:**
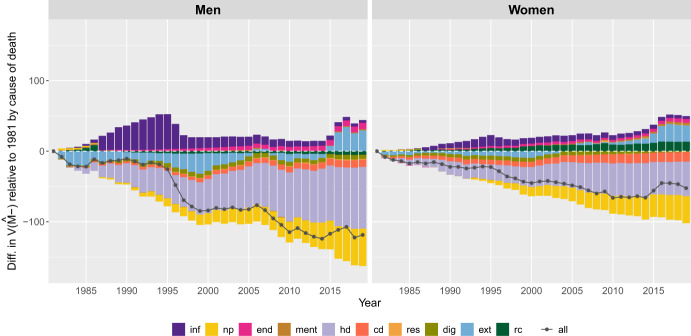
Annual difference in the variance below the mode, $V({\hat{ M}}-)$, relative to 1981, by cause of death for men and women in the U.S. 1981-2019. Note: The acronyms for the causes of death used in the legend stand for: All-causes of death (“all”); Certain infectious diseases (“inf”); Neplasms (“np”); Endocrine, nutritional, and metabolic diseases (“end”); Mental and behavioural diseases (“ment”); Heart diseases (“hd”); Cerebrovascular diseases (“cd”); Acute respiratory diseases (“resp”); Diseases of the digestive system (“dig”); External causes (“ext”); Rest of causes “rc”). Smoothing was based on ages above 10.. Source: HMD (2024)

**Fig. 10:**
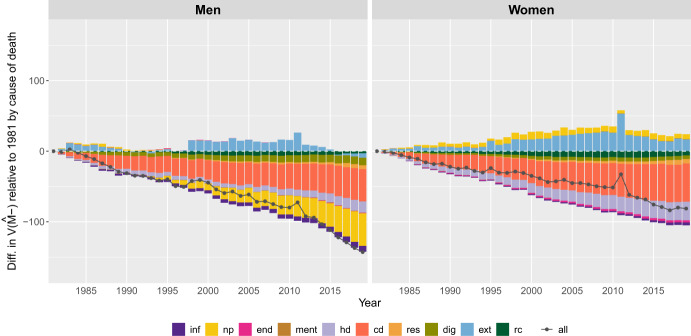
Annual difference in the variance below the mode, $\text V({\hat{\text M}}-)$, relative to 1981, by cause of death for men and women in Japan, 1981-2019. Note: The acronyms for the causes of death used in the legend stand for: All-causes of death (“all”); Certain infectious diseases (“inf”); Neplasms (“np”); Endocrine, nutritional, and metabolic diseases (“end”); Mental and behavioural diseases (“ment”); Heart diseases (“hd”); Cerebrovascular diseases (“cd”); Acute respiratory diseases (“resp”); Diseases of the digestive system (“dig”); External causes (“ext”); Rest of causes “rc”). Smoothing was based on ages above 10. Source: HMD (2024)

**Fig. 11:**
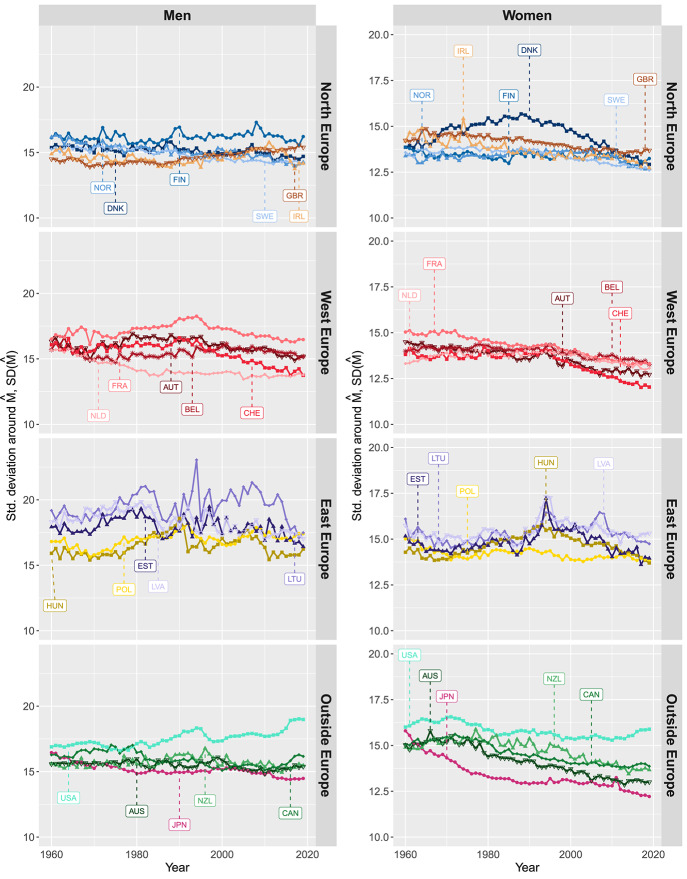
Trends in standard deviation around the mode, $SD({\hat{M}})$, for men and women in several countries in and outside Europe, 1960 to 2019. Note: Smoothing was based on ages above 10. Source: HMD (2024)

**Fig. 12:**
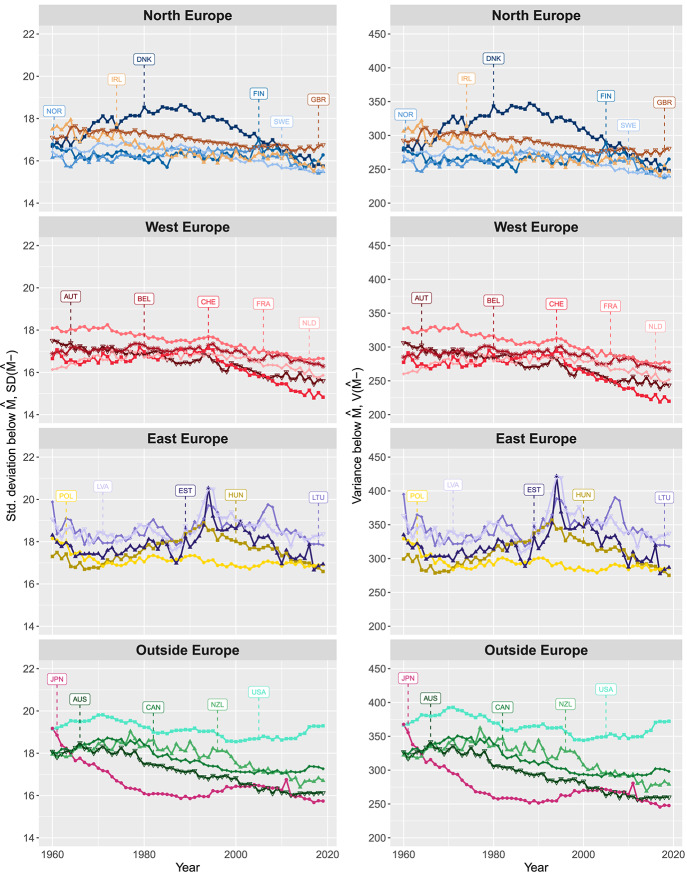
Trends in standard deviation below the mode, $SD({\hat{M}}-)$ and the variance below the mode, $V({\hat{M}}-)$, for women in selected countries in and outside Europe, 1960 to 2019. Note: Smoothing was based on ages above 10. Source: HMD (2024)

**Fig. 13:**
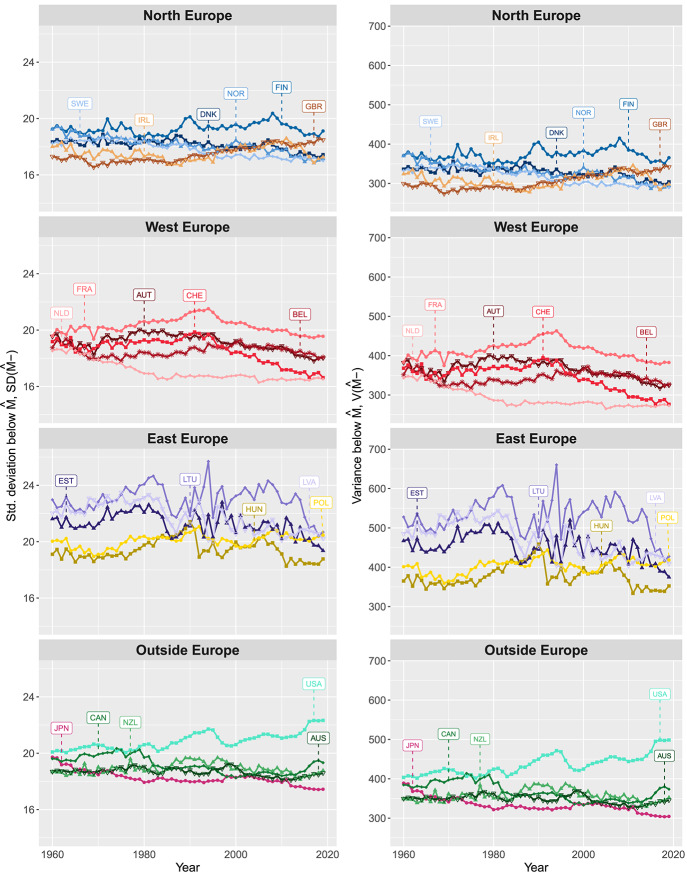
Trends in standard deviation below the mode, $SD({\hat{M}}-)$ and the variance below the mode, $V({\hat{M}}-)$, for men in selected countries in and outside Europe, 1960 to 2019. Note: Smoothing was based on ages above 10. Source: HMD (2024)

Figures 9 and 10 illustrate changes in the dispersion of deaths below *M*, measured by $V({\hat{M}} \textit{-})$, by cause of death and sex from 1981 to 2019 in the U.S. and Japan, respectively. Changes are measured relative to the base year of 1981. Since 1981, deaths from all causes combined at ages below *M* have become less dispersed for both Japanese and U.S. men and women. However, the causes of death responsible for this decline vary by country and genders.^2^

In the U.S., the decline in the dispersion of deaths below *M* for men was primarily driven by heart disease and neoplasms (Fig. 9). From the beginning of the study period until 2015, external causes of death also contributed to the reduction in variability of deaths at ages below *M*. Their impact was greater than that of neoplasms until the early 2000s; however there was a gradual decline in subsequent years. Since 2015, external causes have been the main contributor to the increase in the dispersion of deaths at ages below *M*. Cerebrovascular and digestive system diseases, as well as causes grouped under “rest of causes,” contributed to the decline in $SD(M-)$, albeit to a lesser extent. In contrast, infectious diseases, endocrine diseases, mental disorders, and respiratory diseases contributed to the increase in $V({\hat{M}}\text{- })$, with infectious diseases having the highest positive contribution from the late 1980 s to the mid-2010s. However, its contribution has been declining since 1995. While the impact of endocrine diseases, mental health disorders, and respiratory diseases on changes in $V({\hat{M}}\text{- })$ since 1981 was relatively small, the contribution of endocrine diseases have shown an upward trend since 1990.

For women, heart diseases and neoplasms have been the main drivers of the reduction in the dispersion of deaths at ages below *M* since 1981. However, unlike men, cerebrovascular diseases have also played a significant role in this reduction, especially since 2000. Digestive diseases have also contributed to the decline in $V({\hat{M}}-)$, though their impact has been smaller than that of other causes and has become increasingly negligible since 2005. Since 1981, endocrine diseases, infectious diseases and the “rest of causes” category have led to higher $V({\hat{M}}-)$ values, though their contribution has been relatively small. As with men, infectious diseases were the largest contributor from the mid-1980 s to the late 1990 s. The “rest of causes” category has shown an increasing trend since the 1990s and reached its highest positive contribution between 2005 and 2015, when it was surpassed by external causes of death.

For Japanese men, the causes responsible for the decline in $V({\hat{M}}-)$ since 1981 include cerebrovascular diseases and, since the early 2000s, neoplasms (Fig. 10). Other causes, such as heart diseases, digestive diseases, infectious diseases, respiratory diseases, and those classified as “rest of causes,” also contributed to this decline, although to a lesser extent. Since 1981, mental and endocrine diseases have had almost no effect on changes in $V(M-)$.The external causes of death category was the only one that led to an increase in $V({\hat{M}}-)$ since 1981, with the greatest impact between the mid-1990 s and 2015.

Similar to men, cerebrovascular diseases have contributed to the decline in dispersion below the modal age among women since 1981. Heart diseases and the causes grouped as “rest of causes” have also played a role, but heart diseases have contributed more than the “rest of causes” category. Digestive diseases, endocrine diseases, respiratory diseases, and infectious diseases contributed very little to the decline. In contrast, neoplasms and external causes of death were responsible for widening the distribution of ages at death than in 1981.

## Discussion

Our study is the first to document long-term trends in $SD(M-)$ and to compare them with $SD(M+)$ trends across several European and non-European countries since 1960. We also introduce a novel decomposition method, which is the first attempt to quantify the contributions of specific causes of death to changes in $SD(M-)$ over time.. Our results show that variability in ages at death—both above and below the mode—has declined for men and women in all countries included in the study. This indicates a general reduction in the heterogeneity of health outcomes at the population level, suggesting that population health gains are now more evenly shared among individuals. At the individual level, it reflects a decline in uncertainty surrounding the timing of death.

However, these improvements have not occurred uniformly across all ages. This is evidenced by consistently higher levels of $SD(M-)$ compared to $SD(M+)$, indicating larger mortality inequalities at younger than older ages. Moreover, we observed greater cross-country differences in mortality inequality at younger ages, further highlighting the uneven progress in reducing premature mortality across populations. One possible explanation for the variation in countries’ success in reducing premature mortality lies in differences in healthcare coverage, utilization, and access as well as health-related behaviours—disparities that tend to be more pronounced at younger ages. This difference is evident when comparing the Canadian and U.S. healthcare systems. Canada provides universal healthcare coverage for all individuals, while the United States operates a mixed system of public and private insurance, with eligibility depending on factors such as employment, income, and age. For instance, Americans gain broader access to health insurance starting at age 65 through Medicare. A comparison of 18 OECD countries indicates that the United States performs poorly in terms of mortality rates for individuals under the age of 70. However, its ranking improves significantly for individuals over 70. This trend can be attributed to factors such as broader access to health insurance through Medicare for individuals aged 65 and older, as well as more aggressive treatments and higher medication use for elderly patients with chronic diseases in the U.S. compared to other countries (Ho & Preston, 2010). Another possible explanation for cross-country disparities in premature mortality inequalities may lie in differences in health-related behaviors such as smoking, alcohol use, and obesity. For example, among the countries included in this study, Hungary, Denmark, and Poland recorded the highest estimated smoking-related death rates in 2019, with approximately 82 and 71 deaths per 100,000 people, respectively. In the early 1990 s, Denmark, Ireland, and Poland had the highest rates, at around 180, 177, and 161 deaths per 100,000 people, respectively (IHME, 2024).

The greater variability in deaths below the modal age, compared to those above it, along with the differing declining patterns in $SD(M-)$ across countries, provided the rationale for focusing our decomposition analysis on $SD(M-)$ rather than $SD(M+)$. Therefore, to better understand the factors responsible for changes in $SD(M-)$ over time, we used a double decomposition approach to assess the contribution of causes of death to these changes. First, we separated the changes in $SD(M-)$ into those due solely to shifts in *M* (the “mode” component) and those due to changes in the age-at-death distribution (the “distribution” component). Next, we decomposed the “distribution” component by cause of death to identify the specific causes contributing to changes in $SD(M-)$. We applied this double decomposition approach to Japan and the U.S.

In the U.S., the overall decline in the all-cause dispersion of deaths at ages below the mode death since the early 1980 s was primarily driven by heart diseases and neoplasms. However, this decline was partially offset by infectious diseases, particularly among men, between 1985 and 2000, as well as by external causes after 2015. Similarly, in Japan, the variability of deaths at ages below *M* from all-causes combined followed a downward trend for both men and women since 1981. Cerebrovascular diseases, heart diseases (women), and neoplasm (men) contributed the most to this decline. However, their contribution to decreasing the all-cause variability of deaths below *M* has been somewhat offset by external causes of death and neoplasms (in women), which have increased lifespan variability at these ages since the mid-1990 s.

The increase in variability of deaths from infectious diseases at ages below the mode in the U.S. reflects the HIV epidemic, which disproportionately affected men. Additionally, the positive contribution of external causes of death to lifespan inequalities at ages below the mode underscores the crisis known as “deaths of despair” (Case & Deaton, 2021). This term refers to deaths resulting from suicide, drug overdose, and alcohol-related liver disease, which have been particularly common among middle-aged individuals. The reduction in the negative contribution of external causes of death to lifespan variation observed since the late 1990 s may be associated with the onset of this crisis around that time. External causes of death have also been responsible for the increase in the variability of deaths at ages below the mode in Japan since the late 1990 s. This increase may be partly linked to the rise in suicide mortality rates. In fact, Japan’s suicide rates have increased since the economic crisis of 1998, with a more significant increase observed among males than females (Wada & Gilmour, 2016; Wada et al., 2012; Dhungel et al., 2022). In both countries, younger individuals were primarily affected by these mortality crises. Since $SD(M-)$ is designed to focus on premature mortality (deaths that occur before the modal age of death), it is not surprising that our proposed decomposition emphasizes these crises, particularly in the U.S.

Our results contribute to the ongoing debate about the recent stagnation in life expectancy in the United States, suggesting that this stagnation is more likely due to increased mortality from external causes of death (Woolf & Schoomaker, 2019) than from cardiovascular diseases (Mehta et al., 2020). We found that external causes of death were responsible for the increase in the variability of deaths at ages below the mode, while cardiovascular diseases have contributed to reducing this variability. However, it should be noted that our analysis did not include mortality at older ages; therefore, we cannot exclude the possibility that cardiovascular disease mortality also plays a role in the stagnation of U.S. life expectancy. Therefore, the stagnation in life expectancy may be partly explained by the slower decline in cardiovascular mortality at older ages. The selective age range on which our analysis focused, i.e. from age 10 to the modal age at death, *M*, also explains why we do not capture the important role of perinatal and congenital diseases in reducing life expectancy inequality, as Permanyer et al. (2024) did in their study of 10 low-mortality countries. However, our findings regarding neoplasms, which contributed to the increase in lifespan variation among Japanese women, are consistent with those of Permanyer et al. (2024), who observed a similar trend in several Western countries.

On one hand, focusing solely on premature mortality might be seen as a limitation of our proposed double decomposition, as it provides only a partial view of the evolution of lifespan inequality. On the other hand, concentrating on a specific component of overall mortality makes it easier to derive public health interpretations, thereby highlighting potential policy implications. The factors contributing to the decline in premature mortality often differ from those driving reductions in old-age mortality. For instance, declines in premature mortality are frequently linked to improved injury prevention, reduced infectious and external causes of death (such as accidents, violence, or substance-related deaths), and better management of chronic conditions during midlife. Conversely, declines in old-age mortality are more closely associated with advances in medical care, long-term disease management, and healthier ageing. Focusing on a limited age range also reduces heterogeneity and uncertainty in interpreting mortality dynamics. Causes of death at younger ages are typically more distinct and better defined, whereas at older ages, multimorbidity and diagnostic ambiguity increase, making attribution of mortality trends mode complex. Moreover, when all ages are analysed together, the high concentration of deaths at older ages can dominate the overall pattern, potentially obscuring meaningful changes at younger ages where the societal and economic impacts of premature deaths are greatest. Therefore, targeting public health interventions toward the entire population may be less effective than designing strategies tailored to specific age groups or causes of death. Separating $SD(M-)$ and $SD(M+)$ offers an important advantage by helping to identify which segment of the mortality distribution should be prioritised in policy efforts. Since $SD(M-)$ is typically greater than $SD(M+)$ in most countries, governments should prioritise reducing premature mortality, where the potential gains in lifespan equality are greatest. Thus, the double decomposition of $SD(M-)$, which can equally be applied to $SD(M+)$, is a useful tool for identifying the factors that drive the compression, or lack thereof, of premature mortality.

## Figures and Tables

**Fig. 14:**
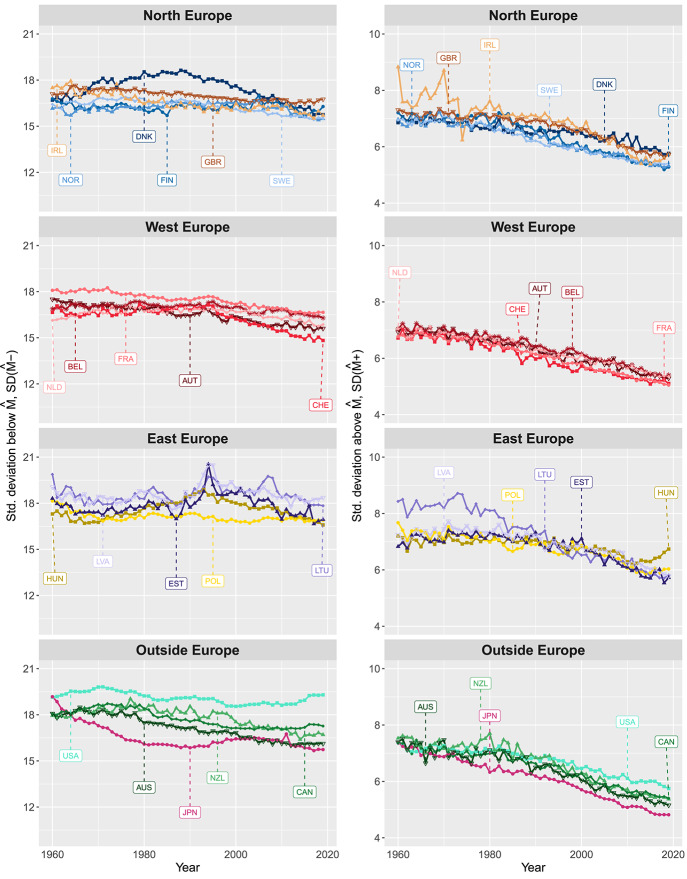
Trends in standard deviation below the mode, $SD({\hat{M}}-)$ and the standard deviation above the mode, $SD({\hat{M}}+)$, for women in selected countries in and outside Europe, 1960 to 2019

**Fig. 15:**
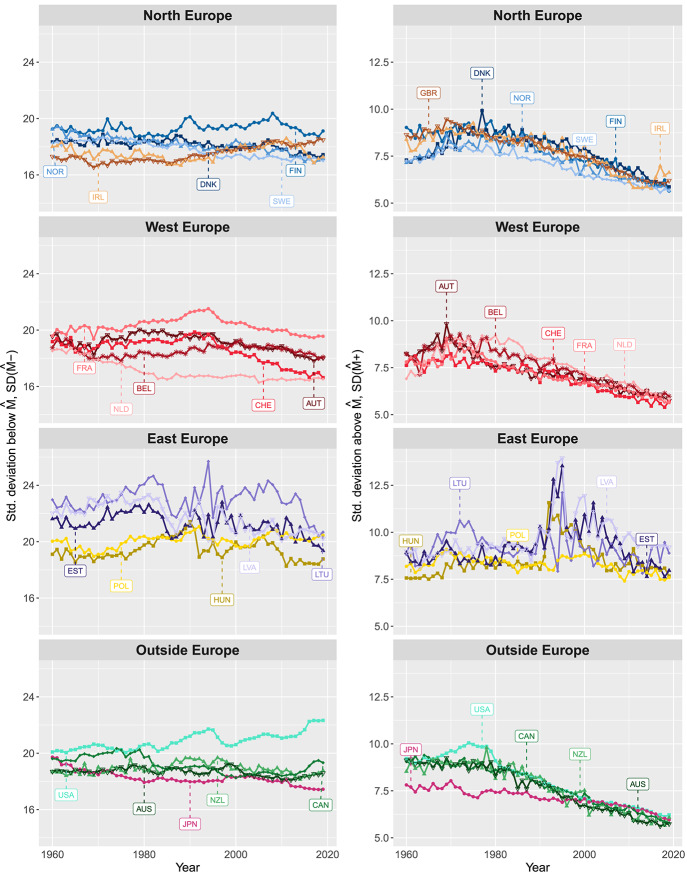
Trends in standard deviation below the mode, $SD({\hat{M}}-)$ and the standard deviation above the mode, $SD({\hat{M}}+)$, for men in selected countries in and outside Europe, 1960 to 2019. Note: Smoothing was based on ages above 10. Source: HMD (2024)

## Data Availability

The datasets analyzed and the R program used to estimate the results of the current study will be made available on GitHub shortly.

## Appendix

See Figures (11, 12, 13, 14, 15).

## Footnotes

1 *f*(*x*) without a superscript represents the all-cause age-at-death distribution.

2 Figs. 9 and 10 show changes in the variance at ages below *M*, $V({\hat{M}}-)$ by cause of death. We have used the variance indicator rather than the standard deviation indicator for the decomposition of the “distribution” component by cause of death because the square root of a difference is not equal to the difference of the square roots. See Sect. 3.2 for more details.
